# Coronavirus pandemic in the Nordic countries: Health policy and economy trade-off

**DOI:** 10.7189/jogh.12.05017

**Published:** 2022-08-08

**Authors:** Furqan B Irfan, Raoul Minetti, Ben Telford, Fahad S Ahmed, Ayesha Y Syed, Nick Hollon, Seth C Brauman, William Cunningham, Mohamed E Awad, Khaled J Saleh, Akbar K Waljee, Nele Brusselaers

**Affiliations:** 1 Institute of Global Health, Michigan State University, East Lansing, Michigan, USA; 2 Department of Neurology and Ophthalmology, College of Osteopathic Medicine, Michigan State University, East Lansing, Michigan, USA; 3 Department of Economics, Michigan State University, Marshall-Adams Hall, East Lansing, Michigan, USA; 4 College of Osteopathic Medicine, Michigan State University, East Lansing, Michigan, USA; 5 Department of Pathology, Wayne State University, Detroit, Michigan, USA; 6 Ferncare Free Clinic, Ferndale, Michigan, USA; 7 University of Michigan Medical School, Institute for Healthcare Policy and Innovation, Ann Arbor, Michigan, USA; 8 University of Michigan Medical School, Department of Internal Medicine, Division of Gastroenterology and Hepatology, Ann Arbor, Michigan, USA; 9 Centre for Translational Microbiome Research, Department of Microbiology, Tumour and Cell Biology, Karolinska Institutet, Stockholm, Sweden; 10 Global Health Institute, Antwerp University, Antwerpen, Wilrijk, Belgium

## Abstract

**Background:**

Countries making up the Nordic region – Denmark, Finland, Iceland, Norway, and Sweden – have minimal socioeconomic, cultural, and geographical differences between them, allowing for a fair comparative analysis of the health policy and economy trade-off in their national approaches towards mitigating the impact of the COVID-19 pandemic.

**Methods:**

This study utilized publicly available COVID-19 data of the Nordic countries from January 2020 to January 3, 2021. COVID-19 epidemiology, public health and health policy, health system capacity, and macroeconomic data were analysed for each Nordic country. Joinpoint regression analysis was performed to identify changes in temporal trends using average monthly percent change (AMPC) and average weekly percent change (AWPC).

**Results:**

Sweden’s health policy, being by far the most relaxed response to COVID-19, was found to have the largest COVID-19 incidence and mortality, and the highest AWPC increases for both indicators (13.5, 95% CI = 5.6, 22.0, *P* < 0.001; 6.3, 95% CI = 3.5, 9.1, *P* < 0.001). Denmark had the highest number of COVID-19 tests per capita, consistent with their approach of increased testing as a preventive strategy for disease transmission. Iceland had the second-highest number of tests per capita due to their mass-testing, contact tracing, quarantine and isolation response. Only Norway had a significant increase in unemployment (AMPC = 2.8%, 95% CI = 0.7-4.9, *P* < 0.009) while the percentage change in real Gross Domestic Product (GDP) was insignificant for all countries.

**Conclusions:**

There was no trade-off between public health policy and economy during the COVID-19 pandemic in the Nordic region. Sweden’s relaxed and delayed COVID-19 health policy response did not benefit the economy in the short term, while leading to disproportionate COVID-19 hospitalizations and mortality.

Various strategies for containing and mitigating the COVID-19 pandemic have been implemented worldwide. Public health and response measures or non-pharmaceutical interventions (NPIs) have been utilized to varying degrees to contain and/or mitigate the pandemic, including face masks and hand hygiene, social distancing, border closures, lockdowns and stay-at-home mandates, closure of non-essential businesses and educational institutions, ban on mass gatherings, quarantine and patient isolation, mass screenings, and contact tracing [1]. The combined effect of the pandemic and the NPIs has resulted in economic impacts and hardship, making countries balance between implementing the NPIs and protecting the economy and livelihood of their populations [2].

The Nordic countries have similar population density, climate and lifestyle, and are among the top twelve developed countries according to the Human Development Index of the United Nations. Around 27 million people live in the Nordic countries, with 38% living in Sweden (10.1 million), 20% in Norway (5.4 million), 21% in Denmark (5.8 million), 20% in Finland (5.5 million) and 1% in Iceland (0.36 million). The limited socioeconomic and cultural differences between and within these countries make them the ideal setting for comparing the effect of different strategies to tackle the COVID-19 pandemic.

In these countries, the first COVID-19 cases were confirmed by the end of January 2020, with the first confirmed deaths occurring between March 11 and 23. While the “Swedish strategy” relied on the citizens’ responsibility for social distancing, working from home, hand washing and limiting non-essential travel, Norway, Finland, and Denmark implemented strict mitigation strategies with the main stated purpose of protecting the elderly and not overwhelming the health care system. Iceland had adopted a strict “test, trace and isolate” approach early on from March 2020 onwards.

The aim of the study was to determine how the Nordic countries have balanced this trade-off between the restrictive COVID-19 health policy and response measures and their impact on the economy. The COVID-19 public health and response measures will be determined for each Nordic country and their effectiveness assessed by the epidemiology of COVID-19, public health and health care capacity, and the community response to the COVID-19 guidelines. The associated negative economic effects will be measured by comparing the macroeconomic indicators and trends for the Nordic region during the pandemic. The generalisability and application of the trade-off in the Nordic countries to comparable regions will provide evidence for better understanding and decision-making, thereby contributing to the science on how to save lives and support the economy during a pandemic.

## METHODS

This study utilized publicly available COVID-19 data of the Nordic countries from January 2020 to January 3, 2021. Epidemiological COVID-19 data (relative numbers adjusted for population size unless otherwise mentioned) including incidence, mortality and case-fatality rate (CFR) were analysed using data sourced from the World Health Organization (WHO) Coronavirus (COVID-19) Dashboard and the European Centre for Disease Prevention and Control [3,4]. The CFR was defined as the number of deaths per confirmed COVID-19 cases (deaths from COVID-19/confirmed COVID-19 cases).

Public health and health system capacity was assessed by COVID-19 testing, test positivity rate [5], hospitalizations, and intensive care unit (ICU) admissions [6]. The test positivity rate was defined as the percentage of all tests conducted with positive results for COVID-19 infection (100 × number of new confirmed cases/number of tests performed) [7].

We also evaluated the health policy measures in the Government Response Stringency Index, a composite measure rescaled to a value from 0 to 100 (100 = strictest response) based on indicators that included: school closures, workplace closures, cancellation of public events, restrictions on public gatherings, closures of public transport, stay-at-home requirements, public information campaigns, restrictions on internal movements, and international travel controls [8,9].

Quantitative data were collected to evaluate the population response to the government’s health policy measures. “Social Distancing” estimates based on change in mobility from anonymous cell phone data from the Institute for Health Metrics and Evaluation (IHME) were also examined [10]. “Mask Use” data defined as “the percentage of the population who say they always wear a mask in public,” was also obtained from IHME [10].

The data from macroeconomic indicators to evaluate the effect of COVID-19 pandemic and health policy control measures on the economy included “Economic growth, percent change in quarterly real GDP” and unemployment rate (monthly), both of which were sourced from Organization for Economic Co-operation and Development (OECD) and the data repository GlobalEconomy.com [11,12].

### Statistical methods

Corrections for populations were performed using 2020 mid-year population for the Nordic countries and standardizing data per 100 000 persons. A descriptive analysis was performed to determine the impact of COVID-19 in the Nordic countries utilizing the following variables: number of new COVID-19 cases, (per 100 000 persons), number of deaths, (per 100 000 persons), case-fatality (in percentage), COVID-19 tests conducted (per 100 000 persons), test positivity (in percentage), COVID-19 hospitalization (per 100 000 persons), COVID-19 intensive care unit (ICU) admissions (per 100 000 population).

Joinpoint regression analysis was performed to identify changes of temporal trends of pandemic development and response indicators. Joinpoint allows for the identification of time points that are associated with statistically significant changes in trends using a Monte Carlo permutation method and calculates the monthly (MPC) and weekly percentage changes (WPC) (weighted least-squares method) for each key indicator. Additionally, the WPCs were weighted by length of interval and averaged to calculate the average weekly percent change (AWPC). The AWPC allows for the use of a single number to describe the average WPCs throughout the course of the year [13]. It is valid even if the joinpoint model indicates that there were changes in trends throughout the year [13]. Analyses were stratified by country and performed using the Joinpoint software (version 4.8.0.1). A *p*-value of <0.05 was considered statistically significant.

We obtained AWPC and their 95% confidence intervals (CI) during the study period, across the Nordic countries for the epidemiological, public health and health system capacity, health policy measures, population response variables. For monthly unemployment rate (in percentage) we obtained average monthly percent change (AMPC). We obtained quarterly data for real GDP growth for which joinpoint regression analysis was not feasible. Instead, quarterly percentage change was calculated for real GDP growth.

## RESULTS

The COVID-19 mitigation strategies utilized by the Nordic countries are summarized in **Table 1**. Between January 2020 and January 3, 2021, there were 715 609 COVID-19 cases and 12 315 deaths in the Nordic region (**Table 2**). Denmark, Finland, and Norway had an initial peak of COVID-19 cases and deaths in April 2020 and then another sustained surge of COVID-19 cases and deaths that started in September and peaked from November 2020 to January 2021. Iceland had the initial peak of COVID-19 cases in March and the second wave of COVID-19 cases peaked from September to October 2020, with COVID-19 mortality surge following the peak of cases by a month. Sweden’s initial surge of COVID-19 cases and deaths was from April to June 2020, with the second surge occurring from November 2020 to January 2021. The CFR of confirmed cases was highest for Sweden (2.2%) followed by Finland (1.5%) and lowest for Iceland (0.5%) among the Nordic countries.

**Table 1:** COVID-19 health policy and response measures utilized by the Nordic countries during 2020

Country/Guiding strategy	Border control	Community engagement	Public health capacity	Public adherence control
Denmark. Four Phase Plan	March-May: International travel restricted	March-May: All public places closed initially, and public gatherings restricted. Business slowly reopened based on need and case numbers. Late reopening of schools. Mask use encouraged.	March-May: Deployment of COVID-19 contact/results mobile applications. Early testing available to first responders and families. Testing of cohorts representing general public begins.	June-August: Police begin monitoring customer volume in local businesses.
		June-August: Reopening of recreational facilities, retail, and private workplaces. Gatherings allowed with limitations of <50 people, increased to 100 people in August.		September-November: Increased police surveillance of businesses. Fines enacted to any persons violating mandates.
	September-November: International travel reopened with testing and quarantine rules established based on country-of-origin risk status. Travelers from low-risk countries not required to follow restrictions.	September-November: Curfews set to food establishments. All other facilities remain open with stricter customer volume control. Gathering restrictions tightened to 50 people in September, decreased to 10 people in October. Masks are required in all open environments and indoors spaces whilst in public.	June-August: Increased testing availability to general public.	December: Enforcement of mandates continued with increased fine amounts.
	December: All travel from the UK closed with few exceptions. All allowed travel required to have negative COVID-19 test within 72 h.	December: Lockdown measures initially lifted in North Jutland. In late December, entire country re-enters lockdown status. All non-essential services closed to the public but can operate from a delivery capacity. Education moved to digital environment. Restrictions extended into new year.	December: Increased testing of peoples aged 15-25.	
Finland. Three Phase Hybrid Plan	March-May: Citizens of Finland are allowed to return regardless of country of departure. Quarantine required for anyone returning from a country with >25 cases.	March-May: Lockdown in effect, all non-essential business closed. All restaurants closed in April. Early childhood care remains open for children of front-line workers. Gyms, and secondary education buildings reopened late May. Gatherings of >10 people are banned.	June-August: Contact tracing mobile applications available for download.	September-November: Regions given a ranking based on risk level. Mandates in effect based on risk level.
		June-August: Remote work mandate removed. Restriction lightened on restaurants. Gatherings of >50 people allowed. Mask use urged but not required. Masks provided free of charge to at risk populations.	September-November: Increased access to testing in accelerated formats based on region-by-region caseloads	December: New resolutions based to enforce mandates to curb transmission. New policy under way regarding vaccine admission.
	December: All travel from UK banned	September-November: New restrictions applied to food and alcohol sale hours. Reinstating remote work from home orders. Education restrictions and bans on gatherings placed on region-by-region basis		
Iceland. Mass-testing, contact tracing, quarantine and isolation response	June-August: All travellers who have spent >24 h in high-risk infection areas must submit 2 tests at border entry, separated by 5 d of quarantine between testing.	March-May: Educational facilities temporarily closed. Reopened with restrictions. Gatherings restricted to <100 people, reduced to <20 people by April. Two-meter proximity rule established; people required to wear masks if within 2m of each other.	March-May: Mobile testing facilities distributed throughout country.	March-May: Public prosecutor sends instructions for fine collection of those in breach of mandates.
		June-August: Any business that cannot follow 2m rule must close. Gatherings of <500 people allowed. Mask not recommended unless cannot follow 2m proximity rule. Gatherings tightened to <100 people by August. End of August public required to follow either 2m rule or wear a mask.		
		September-November: Educational facilities open, all people required to wear masks while in building. The 2m rule reduced to 1m. Gathering ban reduced to <200 people. Gathering ban increased to <10 people in October. 2m rule re-established for all metropolitan areas. Recreational facilities, bars, restaurants closed in October. Maximum classroom capacity set to <50 students.		
	September-November: Free testing provided at all airports for allowed travelers. If no test is documented, 2-week quarantine required. Travelers with documented negative test allowed entry without quarantine.	December: Reopening of all business services with restrictions in place. 20 staff members are now allowed in single space first education schools. 1 staff member and 30 students allowed in secondary education facilities. Masks required in schools when 2m rule cannot be applied.		
	December: All border crossing requirements under review and revisions starting in the new year.		June-August: Increased funding and resource allocation to telehealth services.	June-August: Travelers required to pay for 2 tests and quarantine to enter the country.
Norway. Non-specific reactive response	March-May: Borders closed to all but those with residency permits. Foreigners allowed to leave country.	March-May: All service providers, lifestyle centres, and food business closed. Ban of all gatherings. 1m proximity rule outdoors, 2m proximity rule indoors established. Digital education started. Day-care remains available to children of frontline workers.	No specifics provided	March-May: Fines up to 20000 NOK for anyone in breach of mandates.
	September-November: Travelers must quarantine for 10 d. Allowed to quarantine in local hotels with proper documentation.	September-November: Service providers allowed to operate with restricted customer volume. Ban of >10 people gatherings in private residence.		
	December: All flights from the UK banned. All allowed travellers required to undergo testing at arrival.	December: Gathering restrictions: 5 in houses, 20 in public, 50 in locations without fixed seats, 200 in locations with fixed seats		
Sweden. Reliance on public self-control	March-May: No quarantine requirements for travellers entering Sweden with exception of UK. Travel out of country allowed.	March-May: Upper-level educational schools closed until May; lower-level education remains mandatory. Restaurants must space guests apart. Ban on gatherings of <500 people. Public encouraged to take personal responsibility for well-being.	March-May: Testing available to the public at personal request and expense.	March-May: Breaking of any mandates will result in fines or 6 mo of imprisonment (no documented enforcement). June-August: New plan for public awareness and outbreak control discussed amongst multiple government entities.
	September-November: Extension of ban of travellers from the UK, ban of entry from Denmark.	September-November: End of November, food businesses limit parties to <8 people, with 1m distance between parties. Public gatherings limited to <8 people per party, encouraged to follow similar rule for private gatherings.	June-August: Rapid increases in COVID-19 testing provided to the public at request.	
	December: Continued extension of travel bans in place			

**Table 2:** Population, economy and COVID-19 epidemiology indicators from January 2020 to January 3, 2021

	Denmark	Finland	Iceland	Norway	Sweden
Population (mid-year 2020)	5 792 202	5 540 720	341 243	5 421 241	10 099 265
GDP per capita, current US dollars	60 244	50 926	53 611	62 650	55 065
Life expectancy at birth (years)	79.6	79.4	81.7	81.6	80.7
Population ages 65 and above, percent of total	20.16	22.55	15.62	17.53	20.33
Female population, percent of total	50.29	50.68	49.77	49.46	49.91
Human Development Index (0-1)	0.94	0.938	0.949	0.957	0.945
COVID-19 cases (per 100 000 population)	2892.5	660.6	1701.4	890.5	4528.8
COVID-19 deaths (per 100 000 population)	23.2	10.1	8.5	8.0	98.5
Case-fatality (%)	0.8%	1.5%	0.5%	0.9%	2.2%
COVID-19 tested (per 100 000 population)	182 605	45 317	125 957	56 294	45 955
Test positivity (%)	1.6%	1.5%	1.4%	1.6%	9.9%
COVID-19 hospitalization (per 100 000 population)	967	414	Data not available	391	3,191
COVID-19 ICU (per 100 000 population)	177	79	Data not available	77	546

**Figure 1:**
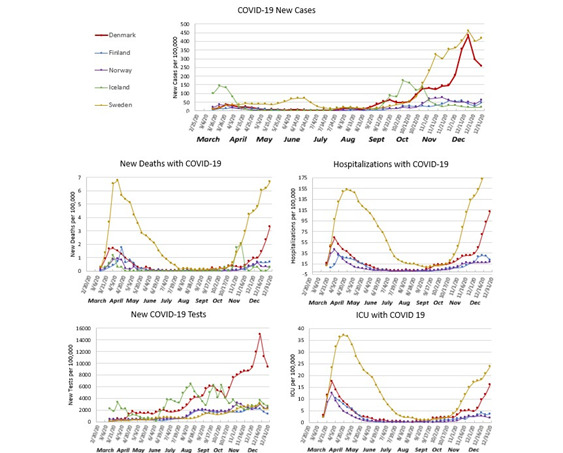
Incidence of COVID-19, deaths with COVID-19, number of COVID-19 tests, hospitalization with COVID-19, ICU hospitalizations with COVID-19, from January 2020 to January 3, 2020.

The country with the highest number of COVID-19 tests per 100 000 persons was Denmark, followed by Iceland. Iceland started large-scale testing the earliest, in March 2020, with sustained effort throughout the study period; Denmark had a gradual increase in COVID-19 tests starting in April and peaking in December 2020; Norway and Finland ramped up testing in July 2020; Sweden was the last to have an increase in testing during September 2020 (**Figure 1**). The overall test positivity was highest for Sweden (9.9%) with comparable numbers for the remaining countries. Sweden had the highest hospitalizations and ICU admissions, followed by Denmark, with comparable numbers for Finland and Norway (**Table 2**).

**Figure 2:**
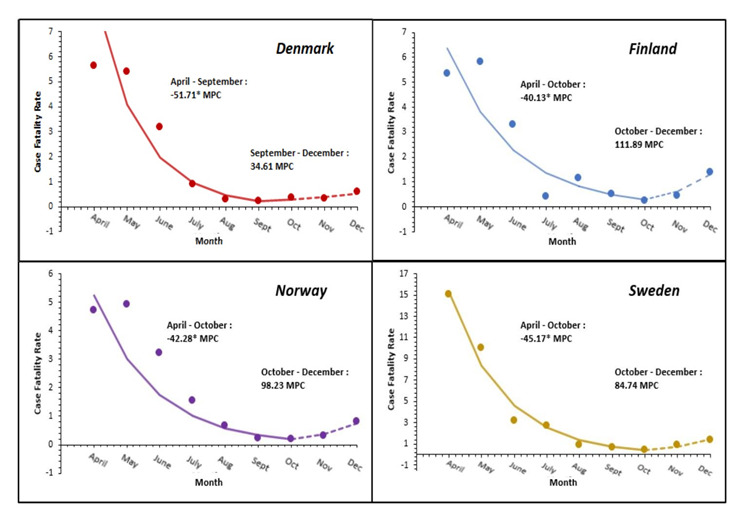
Average monthly percent changes in CFR per country.

**Figure 3:**
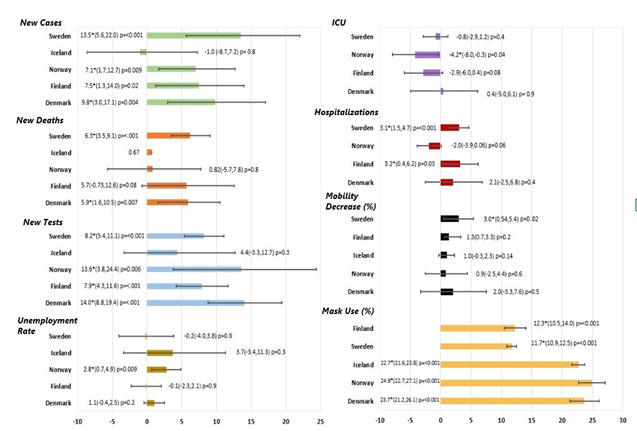
The average weekly percent change (AWPC) for all COVID-19 indicators except unemployment rate. Unemployment rate is displayed as average monthly percent change (AMPC).

The joinpoint regression analysis for CFR was calculated and analysed from April through December 2020. Iceland was excluded from the CFR analysis due to only 29 deaths being reported throughout the year. Overall, the CFR showed a statistically significant decrease in all countries analysed between April and September 2020, with Denmark showing the greatest decrease (MPC = -51.71%, *P* < 0.05) (**Figure 2**). The overall joinpoint regression analysis of the epidemiology, public health and health system capacity indicators during the study period were reported in **Figure 3** using their respective AWPC, its 95% CIs and *p*-values. Denmark, Finland, Norway, and Sweden saw significant increases in their new COVID-19 case AWPC, of which Sweden saw the largest average increase with an AWPC of 13.5% (95% CI = 5.6, 22.0, *P* < 0.001) (**Figure 3**). All countries showed positive COVID-19 mortality AWPC, with Denmark and Sweden’s being considered significant. Of the two countries, Sweden had the largest AWPC (AWPC = 6.3%, 95% CI = 3.5, 9.1, *P* < 0.001) (**Figure 3**). COVID-19 tests AWPC were significantly positive in all countries except Iceland. Denmark had the largest significant positive AWPC of COVID-19 tests administered (AWPC = 14.0%, 95% CI = 8.82, 19.5, *P* < 0.001) (**Figure 3**). Denmark, Finland and Sweden showed an increase in hospitalization; Finland (AWPC = 3.2%, 95% CI = 0.4, 6.2, *P* < 0.05) and Sweden’s (AWPC = 3.1%, 95% CI = 1.5, 4.7, *P* < 0.001) increases were significant and comparable. For ICU occupancy, only Norway showed a statistically significant decrease in AWPC (AWPC = -4.2%, 95% CI = -8.0, -0.3, *P* = 0.04). Decrease in percent mobility was analysed from March to the end of December 2020 for all countries. Each country saw a decrease in mobility; however, only Sweden’s was statistically significant (AWPC = 3.0%, 95% CI = 0.54, 5.43, *P* = 0.02). Mask usage was analysed from February to the end of December 2020 for all countries; all countries saw a significant increase in AWPCs, with Norway having the largest increase (AWPC = 24.9%, 95% CI = 22.7, 27.1, *P* < 0.05) (**Figure 3**).

Unemployment data was reported monthly for each country and analysed to determine the AMPC. However, only Norway had an overall significant unemployment AMPC over the study period (AMPC = 2.8%, 95% CI = 0.7, 4.9%, *P* < 0.009) (**Figure 3**). The percentage change of real GDP was analysed using a non-logarithmic joinpoint regression of quarterly data which yielded the regression slope of each country. The percentage change of real GDP Trends for all countries were determined to be nonsignificant.

## DISCUSSION

**Figure 4:**
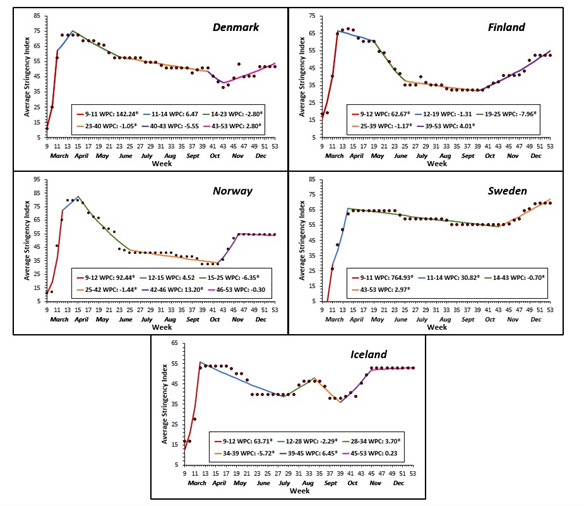
Average weekly percent change (AWPC) in Government Response Stringency Index per country.

**Figure 5:**
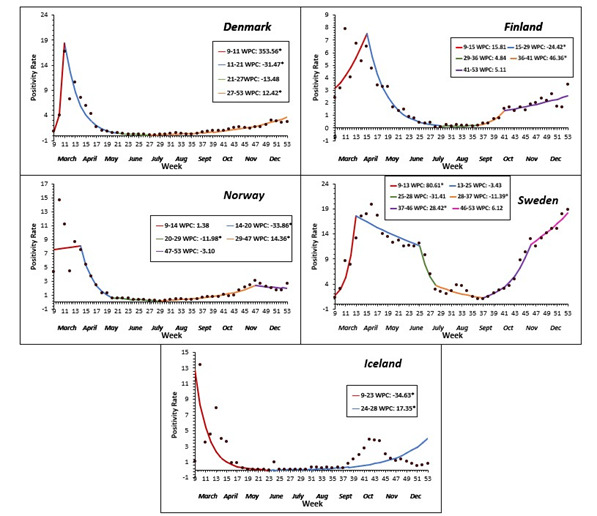
Average weekly percent changes in Test Positivity per country.

To our knowledge, this is the first study of its kind that performed an overall comparative regional analysis on COVID-19 health policy measures, epidemiology indicators and health care utilization, and macroeconomic outcomes. All Nordic countries, except Sweden, implemented relatively strict measures such as lockdowns early in the pandemic, and invested in testing, tracing, quarantine, and other protective measures to limit importing infections and spread within the country (**Table 1**). Sweden’s health policy did not include lockdowns, border controls or quarantine; there were no mandatory restriction measures to limit the number of people in public places, and until early December 2020, Sweden did not recommend face mask usage in public [14]. In contrast, Norway, Denmark, and Finland had strict measures with lockdowns, border controls and quarantine, as early as March 2020 [15-17]. Iceland did not have a lockdown, but instead relied on an aggressive strategy of mass-testing, contact tracing, quarantine, and isolation [18] Denmark’s response was to “Act fast and act with force”; mitigation measures were implemented, and testing capacity was increased nationwide as a preventive strategy to decrease transmission [19,20]. This was also shown by Government Response Stringency Index (**Figure 4**) that had Norway, Denmark, Iceland, and Finland implement a strong initial response in March which was further strengthened in April and decreased gradually in response to decreasing test positivity (**Figure 5**). Sweden had a near static (WPC = -0.7, *P* < 0.05) response from April to mid-October 2020, despite large fluctuations in test positivity (**Figure 4**).

Among all Nordic countries, the number of cases and mortality per capita, as well the number of hospitalizations and ICU admissions were highest in Sweden. Case fatality was 0.9% on average (0.5%-5%) for the other Nordic countries, and 2.2% for Sweden. The test positivity ratio was around 10% for Sweden, and below 2% in all other Nordic countries (in line with other reports), also indicating restricted testing in Sweden and probably the largest proportion of un-diagnosed cases in the Nordic region [20]. These results are similar to other COVID-19 studies in Nordic countries, including a comparative analysis of epidemiological indicators that included Sweden, Norway, Denmark, and Finland [20]. The highest number of COVID-19 tests was carried out in Denmark and Iceland, which correlated with their COVID-19 mitigation strategies. A recent study reported that Denmark had the highest number of COVID-19 tests – 3-4 times higher than its neighbours, which aligns to the findings in this study [20]. All countries had a significant AWPC increase in testing, except Iceland, which implemented a mass-testing strategy from the beginning. The AWPC increases in hospitalizations were comparable for Finland and Sweden, although total hospitalizations for Sweden (3191 per 100 000) were much higher than Finland (414 per 100 000). Only Norway had a significant decline in ICU hospitalizations (AWPC = -4.2%; 95% CI = -8.0, -2.9, *P* = 0.04). This is comparable to a recent study that showed Sweden had the highest number of ICU hospitalizations [17]. Existing health system capacity was critical: pre-pandemic Sweden had the lowest number of ICU beds per capita that were all occupied by April 25, 2020, and ICU beds were rapidly scaled while health systems across the country were being overwhelmed [20]. The community response showed a significant increase in the percentage of population with mask usage across all countries, with Sweden having the lowest average AWPC increase (AWPC = 11.7%, 95% CI = 10.9, 12.5, *P* < 0.001). Interestingly, while mask usage was the lowest, the Swedish population was the only one to exhibit an overall significant decrease in mobility [21,22]. All Nordic countries demonstrated an overall gradually declining mobility trend over the first 27 weeks (except for Finland) that was followed by a variable return to the baseline similar to other reports. The initial decline followed by an increase in mobility had the net effect of cancelling each other out, giving us the minimal non-significant changes in mobility. Sweden's initial decline was approximately half of what we saw in the other Nordic countries which accounted for significant results.

From the macroeconomic perspective, the results do not reveal clear-cut benefits of the looser health policies adopted in Sweden when compared to the rest of the Nordic countries. For example, the trend in percentage change of real GDP was statistically insignificant for all Nordic countries. Compared with its Finnish and Danish neighbours, Sweden exhibits similar dynamics of the unemployment rate. Among these countries, only Norway (AMPC = 2.8%, 95% CI = 0.7, 4.9%, *P* < 0.009) performed significantly worse than Sweden in terms of unemployment. The similar macroeconomic performance of Sweden compared to other Nordic countries suggests that more stringent pandemic mitigation policies and containment can entail limited macroeconomic costs in the short run while enabling the aggregate economy to recover faster from a pandemic. Put differently, the results suggest that there is no trade-off between the restrictive COVID-19 policies to contain the COVID-19 pandemic and macroeconomic outcomes in the Nordic countries.

**Figure 6:**
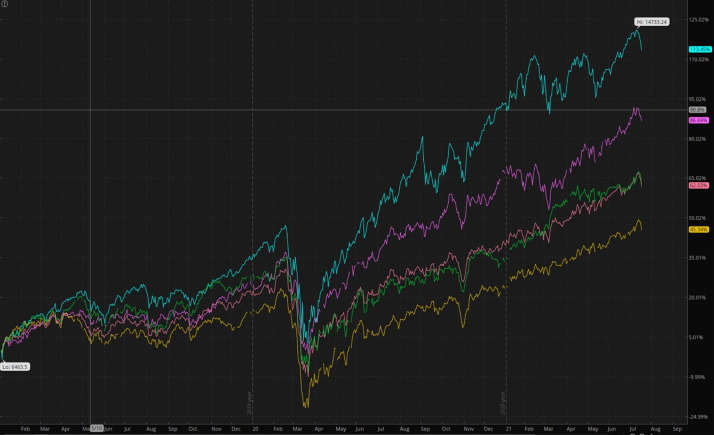
Graph showing comparison of the different stock index’s (January 2020 – July 2021) of each of the Nordic countries. Legend: Yellow: OMXH25 – Helsinki 25 index – Top 25 companies trading in Finland. Green: OMXS30 – Stockholm 30 Index – Top 30 trading in Swede. Red: OMXHN40 – Nordic Top 40 index - Top 40 throughout the Nordic region. Purple: OMXC25 – Copenhagen Top 25 Index – Top 25 trading in Denmark. Cyan: NASDAQ composite – comparison to US market.

The economic profiles of the Nordic countries are similar: they are all open economies dependent on international trade [23]. We thus expect similar exposure to disruptions in international trade and supply chains, market fluctuations, and resource restrictions induced by the COVID-19 pandemic. Domestically, they all feature highly service-oriented economies, despite the relevant industrial exports. As the service sector is the largest and accounts for up to 50% of the economy in some countries, the limitations on social contact, as well as the domestic and foreign travel restrictions, are expected to have long-term impacts on the region’s overall financial health [24]. This study’s one-year data are limited at best, but some broad trends in the percentage change of real GDP and unemployment show that, even with the inclusion of mandatory preventive measures that directly affect the sustainability of economic activity (as opposed to a limited approach), the lack of mandatory monitoring and prevention seems to levy a similar economic recovery timeline. This is comparable to other countries in the region, while costing a disproportionate number of lives to the pandemic, as in the case of Sweden. These conclusions appear to be further validated by the observed patterns of stock market prices in the Nordic countries (**Figure 6**). As stock prices are forward-looking, they can reflect at least to some extent the investors' expectations of the recovery prospects of publicly listed companies. From **Figure 6**, there is no significant difference in the pattern of stock prices in Sweden from January 2020 to July 2021 relative to the stock price pattern in other Nordic countries in the same timeframe. A Nordic (Denmark, Finland, Norway, and Sweden) labour market study up to week 21 (mid-May) of 2020 showed that the labour markets were severely affected, with the Swedish labour market being impacted by a time lag of 2-3 weeks compared to its neighbours, and the cumulative sum of new unemployment and furlough spells for Sweden remaining significantly lower [16]. However, the cumulative sum of new unemployment and furlough spells of Denmark, Finland, and Norway all showed a decreasing trend when compared to Sweden at week 21 (mid-May) of 2020 and would have likely shown an insignificant result had it continued for the entire time span of our study, until the end of 2020 [16].

An economic study determined the cost of COVID-19 mortality in mid-November 2020 to be nearly US$2 trillion (average global value of statistical life ($1.3 million) multiplied by the global number of COVID-19 deaths of 1 400 000), with US$10 trillion loss in the global GDP [25]. Several pandemic-related factors besides health policy have adversely affected the economy, such as behavioural response and economic shocks (labour supply, demand, financial and uncertainty) and connectedness of economies and communities [26]. With globally interconnected economies, supply chains, imports, and exports, an economy cannot be saved in a pandemic when its international trade partners are suffering due to the implementation of COVID-19 health policy measures [27]. In the absence of a trade-off between public health policy and the economy, delaying pandemic response and mitigation measures is not likely to benefit the economy.

Few studies have investigated the trade-off between the COVID-related health policies and the economy [25-28]. Even the studies claiming to report the trade-off are often qualitative or show the impact of higher COVID-19 burden correlating with an economic decline without focusing on health policy measures. Some qualitative evidence is available from the ongoing Evaluation of Science Advice in a Pandemic Emergency (EScAPE) project, a global effort during the COVID-19 pandemic to assess science-based evidence for decision-making, but it does not include hard data to determine trade-offs [29]. A study from the United States (US) showed no trade-off between health policy and economy across the different states, which is consistent with our findings [26]. The United Kingdom had vague, delayed health policies, resulting in a high COVID-19 burden and adverse effects to the economy, relative to neighbouring countries [30-32]. On the other hand, Taiwan, South Korea, and Lithuania had strict and effective health policies earlier in the pandemic, which limited the economic impact and led to low COVID-19 deaths [30,33,34]. We do not find a trade-off in health policy measures and the economy in the Nordic region, and the above studies show that this also holds true for other comparable regions ranging from the US and Europe to East Asia.

Limitations of the study include varying epidemiological definitions and methodologies that were reported by countries in the publicly available COVID-19 databases. The results were based on “recorded” COVID-19 cases, and therefore the “true number” is likely to be higher, depending on the definition used in each country to diagnose an active case. The hospitalization and ICU data for Iceland were not available at the time of analysis. Relying on multiple databases to collect the study variables may have led to timeline differences for data reporting. The macroeconomic indicators had fewer data points compared to the epidemiological indicators, leading to analytic differences, especially for the percent change in GDP. Also, factors affecting the economy besides the mitigation measures were not controlled for in this study.

## CONCLUSIONS

To conclude, this pandemic has demonstrated the variability of COVID-19 mitigation measures across the Nordic countries. Every country has shown a unique reaction, implementing measures based on socioeconomic and geographic factors, epidemiology, health, and politics. Although Sweden had chosen looser COVID-19 health policies to benefit its economy, there is no evidence that this had any short-term economic benefits, while costing disproportionate disease transmission and mortality numbers compared to neighbouring countries.
